# The return of an old nemesis: Survival after severe tricyclic antidepressant toxicity, a case report

**DOI:** 10.1016/j.toxrep.2018.03.009

**Published:** 2018-03-10

**Authors:** Al Giwa, Edwin Oey

**Affiliations:** Department of Emergency Medicine, Icahn School of Medicine at Mount Sinai, One Gustave L. Levy Place, New York, NY, 10029-6574, United States

**Keywords:** Tricyclic antidepressant, Lidocaine, Sodium bicarbonate, Toxicity, Toxicology, Acidosis, Cardiac arrythmia, Torsades, Wide complex tachycardia, Ventricular tachycardia, Recurrent cardiac arrest, Pulseless ventricular tachycardia, VTach, Overdose

## Abstract

Tricyclic antidepressants (TCAs) were first approved by the Food and Drug Administration (FDA) for use as antidepressants in the 1950s. Although their function as an antidepressant in the U.S. has largely been replaced by newer and safer alternatives, they are still prescribed for various conditions, including chronic pain and intractable depression. We will discuss a case of a TCA overdose presenting with generalized tonic-clonic seizures and multiple recurrent cardiac arrests. This is a case of a 34 year-old female who was brought in by Emergency Medical Services (EMS) with generalized tonic clonic seizure, status post intentional ingestion of multiple drugs. Her vital signs were: Temperature-38.8 °C, Heart Rate-140 beats per minute, Respiratory Rate (RR)-25 breaths per minute, Blood Pressure (BP)-139/77 mmHg, Oxygen Saturation (SaO2)-99% on 100% nonrebreather facemask (NRB). Her electrocardiogram (EKG) showed a widened ventricular tachyarrhythmia and she was immediately given an ampule of sodium bicarbonate. Over the span of the subsequent 2 h, she had recurrent pulseless ventricular tachycardic arrest 5 times in the emergency department (ED). After 5 days of further stabilization, the patient had a subsequent complete recovery with normal neurological function at discharge from the medical unit. In the ED it is imperative that we understand the now uncommon presentation of a TCA overdose in order to initiate immediate treatment. It is also important to understand the optimal treatment choices in patients that presents with TCA toxicity, especially arrhythmias that are refractory to initial treatment choices. Overall, severe TCA poisoning is often fatal; however, we demonstrated that with high quality resuscitative efforts, despite multiple arrests, survival to discharge with normal neurological outcome is possible.

## 1 Introduction

Tricyclic antidepressants (TCAs) were one of the first class of commonly prescribed antidepressants to be approved by the Food and Drug Administration (FDA). However, due to severe and potentially fatal side effects, their use in the U.S. has now been replaced by newer and safer alternatives. In the heyday of the use of TCAs, it was not uncommon for emergency department (ED) providers to manage overdoses, which were often recognized by a classic sequela of presenting findings, mostly heralded by the electrocardiogram (EKG). TCAs are still being used for the treatment of various conditions, including depression in patients who have failed other treatments [1]. According to several sources and clinical practice guidelines, TCAs are also utilized in the treatment of chronic pain that is resistant to other modalities [[1], [2], [3], [4], [5]]. As a result there is still a potential for ED providers to see this drug in an overdose. Although a part of all Emergency Medicine (EM) residents’ toxicology training, the now rare presentation of a TCA overdose may not immediately be recognized and the potential for treatment delay is possible. We would like to present a case of a patient who presented with classic signs and symptoms of a TCA overdose despite denying its use.

## 2 Case history

**Fig. 1:**
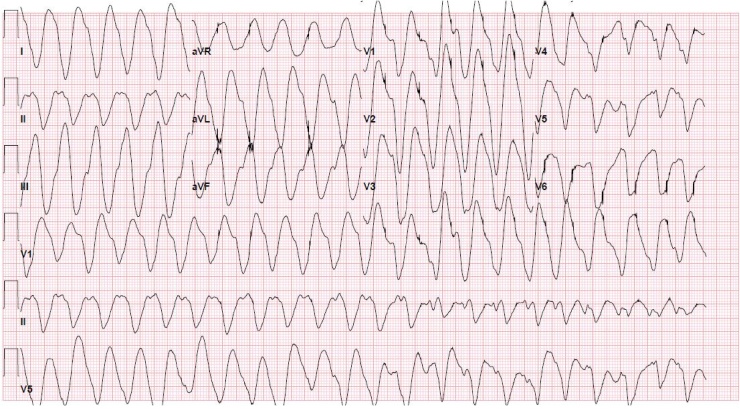
Initial EKG taken as patient arrived. EKG Interpretation: Wide complex tachycardia, concerning for polymorphic ventricular tachycardia. Ventricular rate at 165 BPM, QRS duration prolonged >150 msec.

A 34 year-old female with a past medical history of depression, anxiety, hypertension, and rheumatoid arthritis was brought in by emergency medical services (EMS) after taking an unknown quantity of alprazolam, eszopiclone, and tramadol in an apparent intentional overdose. Per EMS and her “husband” she had been expressing some suicidal ideations, and had a previous history of attempted suicide. She was noted to be actively seizing (generalized tonic clonic) on the EMS stretcher upon arrival to the ED and was immediately given lorazepam 2 mg intravenously (IV) with immediate cessation of the seizure activity. She was transferred to the ED stretcher and was noted to have a Glasgow Coma Scale (GCS) of 8. We prepared to protect her airway via endotracheal intubation, and obtained initial vital signs, as follows: Temp 38.8 °C, heart rate (HR) 140 beats per minute, respiratory rate (RR) 25 breaths per minute, blood pressure (BP) 139/77 mmHg, oxygen saturation (SaO2) 99% on a nonrebreather facemask (Table 1). At this time, an initial EKG (Fig. 1) was taken, which showed a wide complex tachycardia closely resembling ventricular tachycardia (VT). We asked the patient’s significant other for any possible additional medications that she could have ingested, and after initially denying, he recalled she had an “unopened” bottle of nortriptyline that had been lying in a cabinet for several weeks.

**Table 1:** Summary Scheme of Key Events in Correlations to Patient’s Vitals.

Time	Temperature (°C)	BP (mmHg)	HR (beats per minute)	RR (breaths per minute)	SaO2	Key Events and Outcomes
5:27AM	38.8	139/77	140	25	99%	Initial arrival, on nonrebreather. Initial EKG done, see Fig. 1.
5:37AM	38.8	124/82	138	24	99%	Prior to intubation. EKG number 2, see Fig. 2. An ampule of sodium bicarbonate was given along with 2 liters of normal saline.
6:00AM	Unrecorded	112/65	156	20	99%	Post intubation. EKG number 3, see Fig. 3. Bicarbonate drip initiated. Mechanical ventilation initiated and thus RR and SaO2 will remain the same henceforth.
6:10AM	Unrecorded	pulseless VT	pulseless VT	20	99%	First pulseless arrest: defibrillation done. Sodium bicarbonate, epinephrine, magnesium given.
6:19AM	Unrecorded	142/72	134	20	99%	First ROSC, poison control contacted with no further additions.
6:42AM	Unrecorded	pulseless VT	pulseless VT	20	99%	Second pulseless arrest: more rounds of epinephrine, sodium bicarbonate, and calcium gluconate given along with defibrillation.
6:50AM	Unrecorded	64/38	120	20	99%	Second ROSC: Patient became hypotensive, so propofol was discontinued due to its hypotensive effect.
6:54AM	Unrecorded	98/46	131	20	99%	Post second ROSC: hypotension persisted, thus vasopressor support was initiated with appropriate BP response.
6:56AM	Unrecorded	pulseless VT	pulseless VT	20	99%	Third pulseless arrest: more round of epinephrine and sodium bicarbonate given along with defibrillation.
7:04AM	Unrecorded	124/84	124	20	99%	Third ROSC
7:18AM	Unrecorded	pulseless VT	pulseless VT	20	99%	Fourth pulseless arrest: more round of epinephrine and sodium bicarbonate given along with defibrillation.
7:23AM	Unrecorded	118/74	116	20	99%	Fourth ROSC
7:39AM	Unrecorded	pulseless VT	pulseless VT	20	99%	Fifth pulseless arrest: at this point patient was not responding to epinephrine and sodium bicarbonate intravenous pushes, thus decision made to start amiodarone bolus and drip.
7:57AM	38 °C	128/98	122	20	99%	Fifth ROSC. Myoclonic seizure activity noted, propofol restarted.
9:03AM	Unrecorded	138/84	114	20	99%	Transferred to the medical intensive care unit.

**Fig. 2:**
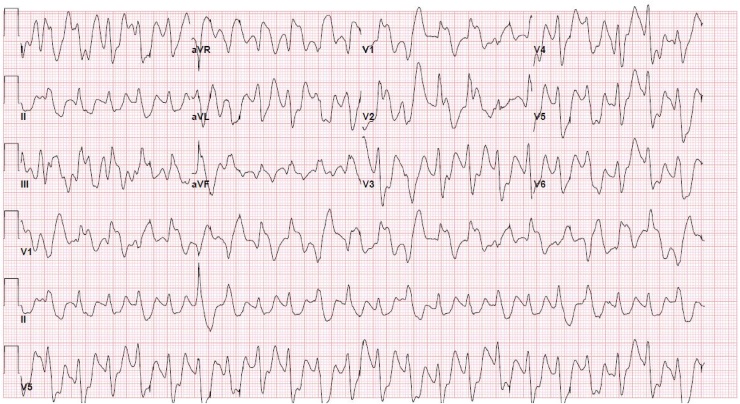
EKG taken post 1 ampule of sodium bicarbonate. EKG Interpretation: Narrowing of QRS intervals is seen, rhythm appears regular, tachycardic, ventricular rate at 153 bpm, QRS prolonged 156 msec, QTc prolonged 619 msec. This EKG shows the immediate effect sodium bicarbonate had in controlling our patient’s wide tachy-arrhythmia.

Her initial venous blood gas at this time was pH 6.94, pCO2 74 mmHg, HCO3 16 mmHg, K 3.8 mEq/L, with a lactate that was >15 mmol/L (higher than the upper test limit). She was immediately given an ampule of sodium bicarbonate (8.4%, 50 mEq) and 2 liters of normal saline 0.9% bolus. A repeat EKG was obtained (Fig. 2), and showed some narrowing of her previously widened QRS. Her vital signs, other than the tachycardia, remained stable. Rapid sequence intubation (RSI) was performed on first attempt with etomidate and rocuronium, and she was maintained on IV infusions of propofol and fentanyl for sedation.

**Fig. 3:**
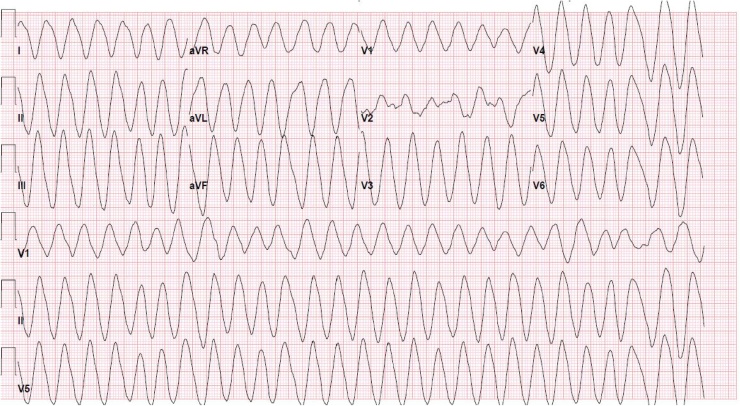
Roughly 5–10 min post intubation, post 2 ampules of sodium bicarbonate with a sodium bicarbonate drip running. EKG Interpretation: Wide complex tachycardia, concerning for Torsade’s like morphology vs sine wave. Ventricular rate 186 bpm, QRS prolonged >150 msec.

Post intubation, the patient was given another ampule of sodium bicarbonate, with a subsequent bicarbonate drip (containing 100mEq of sodium bicarbonate), rectal acetaminophen was ordered, and a nasogastric tube (NGT) was attempted while a third EKG was being obtained (Fig. 3). The EKG at this time showed a return to a wide complex tachycardia with a torsade de pointe like morphology. During the NGT insertion, the patient lost pulses; thus the NGT was aborted and cardiopulmonary resuscitation (CPR) was started with subsequent defibrillation with 200 J and another ampule of sodium bicarbonate, 2 g of magnesium, and two ampules of epinephrine were given before obtaining return of spontaneous circulation (ROSC). Her telemetry strip showed a narrowing of her previous ventricular tachycardia back to a strip similar to Fig. 2. Post ROSC, New York City poison control was contacted, who agreed with our current management, and offered no additional treatment recommendations. Her venous blood gas after the first cardiac arrest revealed a pH of 7.35, pCO2 50 mmHg, HCO3 28 mmHg, K 2.9 mEq/L, lactate 6.5 mmol/L. Her blood pressure remained normotensive.

The NGT was reattempted, however patient’s telemetry rhythm strip at this time widened again to resemble Fig. 3 and she subsequently lost pulses again, and thus the NGT was aborted yet again to restart CPR, defibrillation, with more rounds of both epinephrine, sodium bicarbonate, and calcium before ROSC was regained. During this second arrest, propofol was discontinued as the patient remained hypotensive, and she was given an additional normal saline bolus. Despite ROSC, her hypotension persisted, so peripheral norepinephrine was started with an appropriate response to her blood pressure. Unfortunately, the patient lost pulses three more times, with a total of 5 pulseless ventricular tachycardia arrests in the span of 2 h, each preceded by her cardiac telemetry showing a widened rhythm. Each arrest was followed by further boluses of IV sodium bicarbonate ampules, epinephrine, and defibrillation with ROSC after each arrest. During the final pulseless ventricular tachycardic arrest, 300 mg of amiodarone was administered with a subsequent amiodarone drip.

**Fig. 4:**
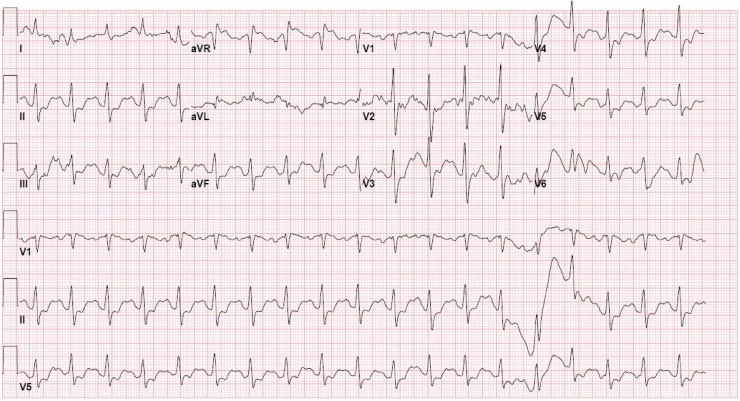
Taken after 5th arrest with successful ROSC. EKG Interpretation: Ventricular rate 115 bpm, QRS narrowed at 128 msec, QTc mildly prolonged at 478 msec, PR interval prolonged at 170 msec. Sinus tachycardia, regular rhythm, QRS narrowed, nonspecific T wave inversions.

After the fifth ROSC, the patient stabilized. Her blood pressure remained stable and normotensive on vasopressor support, her rhythm remained narrow, and her venous blood gas revealed pH 7.49, pCO2 38 mmHg, HCO3 29 mmHg, K 2.6 mEq/L, lactate 6.8 mmol/L. Another EKG (Fig. 4) was taken which showed a stable narrow rhythm. Sodium bicarbonate drip, noriepinephrine drip, and amiodarone drips were maintained. Just as the triple lumen central line was being placed, the patient began to seize, and thus the propofol was restarted. She was also given levetiracetam as recommended by neurology. An NGT was finally placed and activated charcoal was administered in conjunction with the poison control’s updated recommendations. The patient was then transferred to the medical intensive care unit for further care and to initiate Targeted Temperature Management (hypothermia).

## 3 Discussion

TCA poisoning was a common cause of fatal drug overdose in the USA during the era of its frequent prescribed use for the treatment of depression, anxiety, post-traumatic stress disorder (PTSD), and a host of other psychiatric illnesses. It is still being prescribed to patients for a host of other psychiatric and non-psychiatric illnesses [6]. TCAs are rapidly absorbed in the gastrointestinal tract and have a long half-life with an elimination that typically exceeds 24 h [7]. This then leads to a variety of effects on a patient’s body. The most severe of which are cardiovascular and neurological, and typically life-threatening arrhythmias are the final cause of death [7].

Epidemiologically, TCAs have evolved significantly over the last 20 years. Despite newer medications to treat forms of depression and chronic pain, TCAs are still readily used as second or third line agents for refractory treatments [[2], [3], [4], [5], [8]]. Furthermore, TCA overdoses continue to have higher rates of hospitalization and fatality in comparison to other antidepressant agents [[5], [8], [9]]. An analysis between the years 2000–2014 of cases reported to United States poison control centers revealed that although there are numerous different substance exposures, the ones associated with the highest morbidity and mortality has consistently been related to TCA overdose [9]. In 1992, TCA overdose accounted for 1.12 exposures per 10,000 population of the USA [8]. Even if the trend has shifted in the last few years towards utilizing selective serotonin reuptake inhibitors (SSRI) for treatment of depression, the rate of hospitalization has always remained higher in cases of TCA overdose in comparison to SSRI [[5], [8], [9]]. Thus, why it is important for clinicians to understand how TCA overdoses may present and the mainstay of treatment to ensure survival.

The pharmacodynamics of TCAs revolves around four mechanisms. First, it inhibits norepinephrine (NE) reuptake at end nerve terminals. Second, it is a direct alpha-adrenergic blocker. Third, it causes a quinidine-like effect on the myocardium. Finally, it has anticholinergic effects [[7], [10], [11]].

TCA poisoning related cardiovascular toxicity is evident by the presence of arrhythmia and hypotension in patients. Commonly, it causes both tachyarrythmias and bradyarrhythmias, along with a prolongation of the QT interval seen on EKGs and thus, possibly leading to Torsade de pointes [12]. TCA associated wide complex tachyarrythmias that originate from a ventricular origin are commonly ventricular tachycardia and ventricular fibrillation [11]. The QRS prolongation has been a vital role in determining and predicting the severity of TCA poisoning. This is why EKGs are pivotal during an acute TCA poisoning and have become preferable over routine laboratory testing [13]. Bradyarrhythmias also typically occur due to an atrioventricular block. However, the most common arrhythmia to occur due to TCA toxicity is sinus tachycardia and this is due to the anticholinergic properties of TCAs and the inhibition of NE [7].

Hypotension occurs due to a reduction in myocardial contraction and reduced systemic vascular resistance due to the alpha-adrenergic blockade [7]. This is why patients with TCA poisoning may develop acidemia due to a metabolic component from under perfused end organs and eventually respiratory acidosis due to respiratory depression, thus causing a mixed acidotic picture [12]. Overall decrease in myocardial function will inevitably lead to worsening tissue hypoperfusion and hyperlacticemia.

Prolongation of both PR and QT intervals in an EKG with TCA poisoning has also been demonstrated. This is due to blockade within the His-Purkinje system, leading to an intraventricular conduction delay similar to a bundle branch block [14]. TCA poisoning has also been observed to cause Brugada type arrhythmias [14].

The initial treatment for acute TCA poisoning involves a gastric lavage and administering activated charcoal either via a nasogastric tube or oral gastric tube. Typically, this process works best in the first 2 h of ingestion [13]. As our patient was 2–3 h after ingestion, our priority revolved around ensuring her airway and breathing were secured and correcting her cardiac arrhythmias. One should remember that in the “coding patient” status post TCA overdose, there will be decreased splanchnic circulation during the arrest and hence decreased absorption. However, when ROSC is obtained there may be a repeat absorption of the ingested substance, and subsequent repeated systemic effects. Hence, activated charcoal should still be given after the 2 h window has passed.

The treatment for TCA poisoning related cardiac arrhythmia involves multiple steps, including correcting hypoxia, electrolyte abnormalities, hypotension, and/or acidotic states. The current mainstay of treatment utilizes hypertonic sodium bicarbonate therapy as it narrows the QRS complex, improves blood pressure, improves acidemia, and helps control ventricular arrhythmias [[13], [15], [16]]. Furthermore, sodium bicarbonate has shown that it may resolve ventricular arrhythmias even in the absence of an acidotic state [13].

If sodium bicarbonate fails in stabilizing an acute TCA poisoning, only then should other antiarrhythmic medications be considered. Class Ia (quinidine, procainamide, disopyramide) and class Ic (flecainide) are well known sodium channel blockers; thus, they should be avoided in an acute TCA poisoning as it will slow the conduction velocity and further depress myocardial contractility [7]. Class II antiarrhythmics may also precipitate hypotension and may even lead to cardiac arrest [7]. Class III drugs (amiodarone) may prolong QT interval and increase the overall arrhythmic risk [7]. In our patient’s scenario, the amiodarone drip was eventually discontinued within 3 h in conjunction with a cardiology consultation. The patient was also never put on any other antiarrhythmic agents throughout the rest of her hospitalization due to stabilization and lack of further arrhythmias.

Class Ib antiarrhythmic drugs such as phenytoin and lidocaine have also been studied in regards to refractory TCA poisoning related arrhythmias. Phenytoin and lidocaine both act as sodium channel blockers, but unlike class Ia and Ic agents, they do not depress the initial phase of depolarization in healthy cardiac tissue [17]. Furthermore, class Ib agents dissociate quickly from cardiac sodium channels, which results in a faster recovery time in comparison to TCAs [17]. It is thought that class Ib rapid binding to sodium channel directly displaces slower acting agents from the channel (eg TCAs), leaving more channels unbound and therefore facilitating more cardiac conduction [17]. Although phenytoin acts similarly to lidocaine, studies do not show that it has the same effects as lidocaine in an acute TCA poisoning. This may be postulated from the idea that phenytoin does not directly compete with the same sodium channels as TCAs [[13], [17]]. However, lidocaine has been shown to have better effect during an acute TCA toxicity, which makes it the antiarrhythmic of choice for TCA toxicity related refractory arrhythmias [[13], [17]].

There have also been studies utilizing hypertonic saline in the management of TCA poisoning not responding to sodium bicarbonate therapy, with aims of overwhelming the sodium channel blockade effect of TCAs [18]. However, the results have generally been conflicting and thus further studies are needed [19].

Other studies have shown a possible treatment choice spanning around intravenous lipid emulsion therapy for TCA related cardiotoxicity, however the studies are also conflicting and thus further research is needed [20].

## 4 Conclusion

**Fig. 5:**
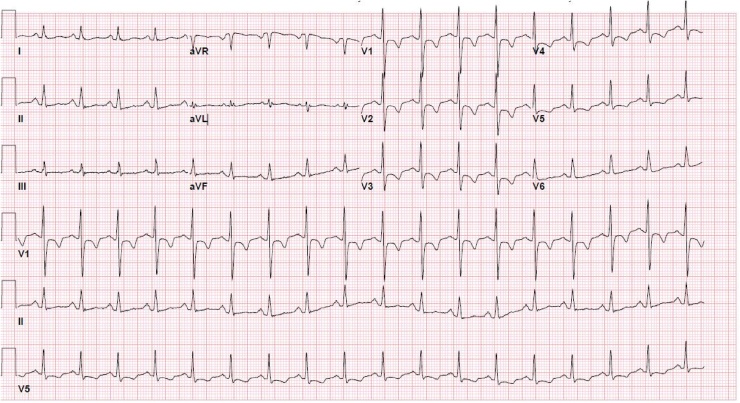
EKG taken prior to patient being discharged, day 5 post ingestion. EKG interpretation: Normal sinus rhythm, mild tachycardia with a ventricular rate of 109 bpm. QRS duration 100 msec and normal. QTc mildly prolonged at 455 msec. PR interval normal at 146 msec. Nonspecific T wave inversions notable over anterior leads.

The patient was extubated after several days of Targeted Temperature Management and continued cardiovascular support. Subsequent evaluations by cardiology and neurology cleared the patient from any prolonged abnormalities or need for long term therapy, as her seizures and cardiac arrest were related to presumed TCA versus tramadol toxicity. Her TCA (Nortriptyline) level was 1581 ng/mL (Table 2), with a therapeutic range of 50–150 ng/mL, which makes us believe that the patient’s symptomatology was mainly related to TCA; however with the understanding that the other medications co-ingested likely worsened the picture, as tramadol is also known for having sodium channel blockade activity. Both laboratory and echocardiography did not reveal any ischemic or structural related causes of her cardiac arrest. Further advanced imaging of her brain with magnetic resonance imaging and an electroencephalography did not reveal any intracranial causes of her seizures. The patient was discharged on day 5 post-arrest with a normal sinus EKG with some T wave inversions, which may be her new baseline (Fig. 5). Her widened QRS and QTc have resolved, however.

**Table 2:** Test Results.

Substance Tested	Results
Alcohol, serum	<10.0 mg/dL
Acetaminophen	<3 mcg/mL
Salicylate	<5 mg/dL
TCA (Nortriptyline)	1581 ng/mL
U-Amphetamine	Negative
U-Barbiturate	Negative
U-Benzodiazepines	Positive
U-Cocaine	Negative
U-Opiate	Negative
U-Phencyclidine	Negative
U-Methadone	Negative
Cannabinoids	Negative

Severe TCA poisoning is often fatal; however, we demonstrated that with high quality resuscitative efforts, despite multiple arrests, survival to discharge with normal neurological outcome could be obtained. Utilization of sodium bicarbonate during these efforts is vital as the first line agent for stabilization, and secondarily utilization of intravenous lidocaine for TCA toxicity should be considered for refractory arrhythmias.

## Conflict of interest

None.
